# Validation of the Italian Multidimensional Psychological Flexibility Inventory Short Form (MPFI-24)

**DOI:** 10.3390/bs16040510

**Published:** 2026-03-29

**Authors:** Giulia Landi, Zhangxuan Bao, Francesco Bruno, Kenneth I. Pakenham, Francesca Chiesi, Eliana Tossani, Silvana Grandi

**Affiliations:** 1 Department of Psychology, University of Bologna, 40126 Bologna, Italy; 2 Laboratory of Psychosomatics and Clinimetrics, Department of Psychology, University of Bologna, 47521 Cesena, Italy; 3 Department of Human and Social Sciences, Universitas Mercatorum, Piazza Mattei 10, 00186 Rome, Italy; 4 School of Psychology, The University of Queensland, Brisbane, QLD 4072, Australia; 5 Department of Neuroscience, Psychology, Drug, and Child’s Health (NEUROFARBA), Section of Psychology, University of Florence, 50121 Florence, Italy

**Keywords:** MPFI-24, scale validation, psychological flexibility, psychological inflexibility, psychometric properties

## Abstract

This research examines the psychometric properties of the Italian Multidimensional Psychological Flexibility Inventory short form (MPFI-24), a measure of psychological flexibility/inflexibility. Study 1 investigated its factor structure, reliability and invariance (across gender, age, and mental health status) based on a dataset comprising 1542 participants (71% female, mean*_age_* = 38.6 years, *SD* = 15.0). Study 2 reexamined the factorial structure in an independent sample (N = 728, 64.88% females, mean*_age_* = 30.94 years, *SD* = 14.07), and assessed both convergent validity (with psychological flexibility/inflexibility measures) and concurrent validity (with distress and well-being measures). Confirmatory factor analyses demonstrated very good fit indices for a first-order model comprising the twelve psychological flexibility and inflexibility sub-processes. In addition, the model structured with two second-order factors—psychological flexibility and inflexibility—each defined by six core sub-processes, showed a good model fit. The Italian MPFI-24 also exhibited strong internal consistency and good convergent and concurrent validity. Measurement invariance was established for gender, age, and mental health status. The Italian MPFI-24 is a psychometrically sound instrument for evaluating psychological flexibility and inflexibility, along with their underlying sub-processes, in an Italian context.

## 1. Introduction

Psychological flexibility refers to the ability to engage with the present moment in a conscious and open way, and to adapt behavior in ways that are consistent with one’s personal values, even when facing psychological challenges (Hayes et al., 2006). From a contextual behavioral science perspective, it is considered a transdiagnostic construct. This construct is at the core of acceptance and commitment therapy (ACT), which identifies six psychological flexibility sub-processes: (1) acceptance, (2) defusion, (3) present moment awareness, (4) self-as-context, (5) values, and (6) committed action (Hayes et al., 2012). Its counterpart is psychological inflexibility, which includes six corresponding opposing sub-processes: (1) experiential avoidance, (2) lack of present moment awareness, (3) self-as-content, (4) fusion, (5) restricted valuing repertoire, and (6) inaction and impulsiveness (Hayes et al., 2012). The central objective of ACT is to strengthen psychological flexibility and thereby foster mental health. Evidence from both field and laboratory studies show that greater psychological flexibility is linked to improved mental health (Gloster et al., 2017; McCracken & Gutiérrez-Martínez, 2011; Young et al., 2022) and higher psychological inflexibility is linked to more psychopathology (Levin et al., 2014; Ong et al., 2024) across diverse populations and settings. A meta-analysis of meta-analytic studies has confirmed the effectiveness of ACT in treating a broad spectrum of mental health problems (Gloster et al., 2020). Furthermore, evidence suggests that ACT brings about therapeutic benefits primarily by fostering psychological flexibility, which serves as the central mechanism of change in the psychological flexibility framework (Stockton et al., 2019).

### 1.1. Measurement of Psychological Flexibility/Inflexibility

The only validated self-report instrument currently available that assesses both psychological flexibility and psychological inflexibility and their corresponding six sub-processes is the Multidimensional Psychological Flexibility Inventory (MPFI) (Rolffs et al., 2018). The MPFI scale consists of 60 items, with the flexibility and inflexibility scales each consisting of 30 items. Five distinct items were designed to assess each flexibility and inflexibility sub-process. Factor analytic studies have confirmed that the MPFI’s factor structure aligns with the theoretical model of psychological flexibility. Specifically, psychological flexibility and inflexibility emerge as two higher order factors, each composed of six distinct first-order sub-processes (Grégoire et al., 2020; Lin et al., 2020; Rolffs et al., 2018; Seidler et al., 2020). Evidence of convergent validity is reflected in the consistent alignment of both global and subscale psychological flexibility and inflexibility scores with established measures designed to assess similar psychological constructs (Rolffs et al., 2018). Empirical studies have supported the discriminant validity of the MPFI by revealing expected-pattern associations between its twelve sub-processes—reflecting psychological flexibility and inflexibility—and conceptually unrelated constructs such as neuroticism, emotional intelligence, inattention, and rumination, as well as with diverse indicators of individual well-being, including psychological distress, relationship satisfaction, and vitality (Rolffs et al., 2018). Evidence for the discriminant validity of the MPFI psychological flexibility scale in relation to distress was provided by a study demonstrating that the six psychological flexibility sub-processes loaded on a psychological flexibility factor, whereas anxiety and depression and a widely used proxy measure of psychological flexibility [Acceptance and Action Questionnaire-II; (Bond et al., 2011)] loaded on a distress factor (Landi et al., 2021a). Results from convergent and discriminative validity analyses revealed that the twelve sub-processes differed in their associations with related constructs and were identified as distinct components capable of functioning independently from one another (Rogge et al., 2019; Rolffs et al., 2018). Additionally, the MPFI has demonstrated sensitivity to change over time (Rolffs et al., 2018), and capacity to distinguish individuals receiving psychological counseling (Rogge et al., 2019), and it has exhibited significant associations with both psychological distress and indicators of individual wellbeing (Rolffs et al., 2018; Stabbe et al., 2019). The MPFI has been translated into various languages and culturally adapted and validated in numerous countries [e.g., Sweden (Tabrizi et al., 2023); Italy (Landi et al., 2021b); Iran (Hekmati et al., 2024); China (Lin et al., 2020), and Japan (Lin et al., 2020)].

The length of the MPFI has however raised concerns about potential response bias affecting reliability (Edwards et al., 2004; Fang et al., 2024) and the burden it places on respondents thereby precluding its use in some studies. Hence, to broaden applicability of the MPFI, Rolffs et al. (2018) used item response theory to develop a shorter version with two items tapping each of the twelve sub-processes, yielding a 24-item measure (MPFI-24). The MPFI-24 preserves the essential theoretical constructs while offering a more efficient format for assessment by the reduced number of items. The MPFI-24 offers a convenient and efficient method for assessing psychological flexibility and inflexibility, particularly in resource-constrained situations such as large-scale epidemiological research or cross-cultural studies (Fang et al., 2024) and time-constrained investigations such as experiential momentary assessment studies or national screening surveys (Grégoire et al., 2020). Evidence indicates that the MPFI-24 subscales effectively assess the twelve psychological flexibility and inflexibility sub-processes (Grégoire et al., 2020; Hekmati et al., 2024). Results of factor analytic studies of the MPFI-24 replicate the MPFI factor structure entailing a second-order structure, characterized by global psychological flexibility and inflexibility, and a first-order factor structure that corresponds with the twelve sub-processes (Fang et al., 2024; Grégoire et al., 2020; Hekmati et al., 2024). This factor structure aligns with the ACT psychological flexibility framework.

The MPFI-24, in its original English form, has demonstrated acceptable levels of internal consistency across various populations, and a slightly lower reliability compared to the parent scale, which is likely attributable to the brevity of each subscale (Grégoire et al., 2020; Rolffs et al., 2018). The MPFI-24 has also been adapted to various cultures and translated into multiple languages, including French (Grégoire et al., 2020), Chinese (Fang et al., 2024), Persian (Hekmati et al., 2024), and Spanish (Barrado-Moreno et al., 2025). Across these adaptations, the MPFI-24 has demonstrated full measurement invariance in relation to mental health status (Hekmati et al., 2024), gender (Fang et al., 2024; Hekmati et al., 2024), and language (Grégoire et al., 2020). The MPFI-24 demonstrated convergent validity through significant associations between its subscales and other conceptually similar measures of psychological flexibility/inflexibility (Barrado-Moreno et al., 2025; Fang et al., 2024; Grégoire et al., 2020; Hekmati et al., 2024). The discriminant validity of the MPFI-24 has also been supported by studies showing its differential associations with positive (i.e., being) and negative (i.e., distress) mental health indicators (Grégoire et al., 2020; Hekmati et al., 2024). Studies have also shown the MPFI-24 psychological flexibility and inflexibility scales demonstrate adequate temporal stability across a range of populations (Fang et al., 2024; Grégoire et al., 2020; Hekmati et al., 2024; Rolffs et al., 2018).

### 1.2. The Present Research

Based on the MPFI Italian validation (Landi et al., 2021b), we conducted two studies designed to evaluate the psychometric performance of the Italian adaptation of the MPFI-24. Study 1 investigated the Italian MPFI-24 factorial structure, reliability and the stability of measurement across demographic and clinical subgroups. Study 2 reexamined the factor structure in an independent sample and assessed the convergent and concurrent validity of the Italian MPFI-24.

## 2. Study 1

Study 1 investigates psychometric characteristics of the Italian version of the MPFI-24 with respect to factor structure, reliability, inter-factor correlations, and measurement invariance across gender, age, and mental health status.

### 2.1. Materials and Methods

#### 2.1.1. Recruitment Procedure and Participants

A total of 1693 Italian-speaking adults (aged 18 and above) took part in an online survey distributed nationwide. Recruitment occurred through WhatsApp and Facebook and a snowball sampling strategy, from July to October 2023. The survey was developed using the cloud-based survey platform (i.e., Qualtrics XM platform, Provo, UT, USA) and because of the recruitment methods used, it was not feasible to calculate an exact response rate. All participants provided informed consent prior to undertaking the survey. The study received ethical approval from the University of Bologna research ethics committee.

The sample included 77.55% (*n* = 1313) females, with a mean age of 33.38 years (*SD* = 13.64; range 18–82). Highest education levels were bachelor’s/master’s degree (*n* = 903; 53.34%), high school (*n* = 720; 42.53%), and middle school (*n* = 70; 4.14%). Regarding employment status, 45.96% (*n* = 778) were employed, 38.04% (*n* = 644) were students, 7.80% (*n* = 132) unemployed, 3.31% (*n* = 56) were retired, and 4.89% (*n* = 83) “other”. Almost half (50.8%, *n* = 860) of the sample was partnered or married, 44.5% were single (*n* = 753), while 4.73% (*n* = 80) were widowed/divorced. Concerning socioeconomic status, 17.25% of the participants (*n* = 295) reported below-average income, 72.89% (*n* = 1234) middle-income, and 9.86% (*n* = 167) above average. Furthermore, nearly one-third of the sample (29.4%, *n* = 498) reported they were currently experiencing psychological or psychiatric challenges, including ongoing therapy and/or medication use.

#### 2.1.2. Measures

**MPFI-24** (Grégoire et al., 2020). The MPFI–24 is a 24-item scale derived from its parent scale (Rolffs et al., 2018). Twelve items each assess psychological flexibility and psychological inflexibility and two assess each of their six constituent sub-processes. Items are scored on a 6-point scale, where 1 indicates “never true” and 6 indicates “always true.” To assess specific sub-processes, scores from pairs of items of each subscale are averaged. Global psychological flexibility and inflexibility scores are calculated by averaging scores across the 12 items for each scale. Higher global psychological flexibility and inflexibility scores reflect higher levels of their underlying construct.

#### 2.1.3. Data Analysis

Most analyses were carried out using SPSS version 29. IBM AMOS 26 with the maximum likelihood estimator was employed to evaluate the factor structure of the instrument, with three first-order confirmatory factor analyses (CFAs)—a one-factor, two-factor, and twelve-factor models—and two second-order CFAs—one with a single higher-order factor and another with two higher-order factors. Following best practices in CFA, we relied on the following indices: Comparative Fit Index (CFI), Chi-Square statistics (χ^2^), Tucker–Lewis Index (TLI) and Root Mean Square Error of Approximation (RMSEA) (T. A. Brown, 2015) to evaluate model adequacy. Values above 0.90 of the CFI and TLI, and values below or equal to 0.08 of RMSEA were interpreted as good model fits (Hu & Bentler, 1999). Akaike Information Criterion (AIC) and Bayesian Information Criterion (BIC) were used to compare and select the optimal model, with lower values reflecting improved fit (Cai & Monroe, 2014). Reliability was assessed using McDonald omega and the relative 95% CI, with values of 0.7 or above considered indicative of acceptable reliability (Ellis, 2010).

Gender, age, and mental health status measurement invariance was examined using Multi-group CFA. Following common practice for examining measurement invariance (Millsap, 2012), we treated age categorically by creating two subsamples based on the median. First, configural invariance was assessed by allowing all model parameters to be freely estimated, ensuring that the basic factor structure was comparable across groups. Second, metric invariance was evaluated by constraining factor loadings to be equal across groups and comparing the model fit to the configural model. Third, scalar invariance was tested by imposing equality constraints on both factor loadings and item intercepts, with its fit compared to the precedent model. Finally, residual invariance was examined by constraining factor loadings, intercepts, and residual variances simultaneously across groups. To determine measurement invariance, we used criteria that are widely recognized in the literature (Kimber et al., 2015; Kim et al., 2017; Rutkowski & Svetina, 2017). Following established guidelines, measurement invariance was supported when observed changes in model fit indices did not exceed the conventional cut-offs of ΔCFI ≤ 0.01, ΔTLI ≤ 0.01, and ΔRMSEA ≤ 0.015. These criteria were adopted as they are less influenced by sample size and model complexity than the χ^2^ criterion (Swami et al., 2023), which is also reported for each model.

### 2.2. Results

#### 2.2.1. Italian MPFI-24 Descriptives

Means, SDs, skewness and kurtosis for each Italian MPFI-24 item are presented in Table S1 of Supplementary Materials. The observed item responses covered the full scale range, with item ratings distributed evenly across the numerical rating scale, suggesting an approximately normal distribution. These indices thereby supported the use of the maximum likelihood estimator for CFAs.

#### 2.2.2. Factor Structure of the Italian MPFI-24

To evaluate the factor structure of the Italian MPFI-24, five models were tested. The first was a one-factor model, assuming that all items reflect a single overarching factor. The second model proposed a two-factor model, in which items were loaded on one of two overarching factors, psychological flexibility or psychological inflexibility. The third model treated each of the twelve sub-processes represented by the subscales of the MPFI-24 as distinct first-order factors. The fourth and fifth models were the one-factor second-order structure which included twelve first-order factors (i.e., the MPFI-24 subscales) and the two-factor second-order model including six first-order factors (i.e., the MPFI-24 subscales) for each of the second-order factors (i.e., psychological flexibility and psychological inflexibility). Although the one- and two-factor models demonstrated poor model fit, the twelve-factor model showed very good fit indices χ^2^ = 565.71, χ^2^/df = 2.92; CFI = 0.98; TLI = 0.98; RMSEA = 0.034; RMSEA CI = [0.030, 0.037]; SRMR = 0.06 (see Table 1).

Fit indices for the second-order model were not adequate for the one-factor model, but they were good for the two-factor model. Strong internal consistency was observed for the global scales of psychological flexibility and inflexibility, as reflected by McDonald omega values of 0.90 and 0.86, respectively. Internal consistency for the twelve sub-process scales varied from 0.71 to 0.92, indicating acceptable to high reliability. Factor loadings for the twelve-factor structure are displayed in Table 2.

#### 2.2.3. Intercorrelations Among the Italian MPFI-24 Factors

Table 3 summarizes the intercorrelations among the Italian MPFI-24 subscales. Significant positive correlations were evinced between global psychological flexibility and all six flexibility sub-processes, with coefficients in the large range (0.67–0.81, *p* < 0.001). All sub-processes within the psychological flexibility domain were positively and significantly correlated, with the majority of intercorrelations falling within the moderate to large magnitude (range 0.32–0.62, *p* < 0.001). Likewise, global psychological inflexibility was moderately associated with all corresponding sub-processes (range 0.32–0.80, *p* < 0.001), which also demonstrated significant positive intercorrelations, with coefficients ranging from moderate to large (range 0.41–0.74, *p* < 0.001), except for experiential avoidance. It had small positive associations with the lack of contact with the present moment (*r* = 0.12, *p* < 0.001) and self-as-content (*r* = 0.19, *p* < 0.001), but was not significantly associated with the remaining psychological inflexibility sub-processes. Correlations between global and sub-process scores of psychological flexibility and inflexibility were generally negative, significant and ranged from small to moderate in magnitude (range −0.05–−0.53, *p* < 0.05). Contrary to expectations, experiential avoidance was weakly but significantly correlated with three out of six flexibility sub-processes (range = 0.05–0.11, *p* < 0.05) and had a non-significant positive relationship with the global psychological flexibility score (*r* = 0.02, ns).

#### 2.2.4. Measurement Invariance

Table S2 of Supplementary Materials reports findings on measurement invariance of the Italian MPFI-24. Specifically, we compared male (*n* = 366, age range 18–81 years, *M* = 35.08, *SD* = 14.52) vs. female (*n* = 1313, age range 18–82 years, *M* = 32.97, *SD* = 13.38) samples, younger (*n* = 842, 79% females, age range 18 –26 years, *M* = 23.30, *SD* = 2.25) vs. older (*n* = 843, 76% females, age range 27–82 years, *M* = 43.46, *SD* = 12.81) samples, and clinical (*n* = 498, 83% females, age range 18–82 years, *M* = 30.93, *SD* = 11.16) vs. community (*n* = 1195, 75% females, age range 18–81 years, *M* = 34.40, *SD* = 14.44) samples. The fit indices of each tested model (configural, metric, scalar, and residual) were adequate. All model comparisons showed values of ΔCFI and ΔTLI below or equal to 0.01, and values of ΔRMSEA below or equal to 0.015. Thus, evidence indicates that invariance for the Italian MPFI-24 holds across gender, age, and mental health status.

### 2.3. Discussion

CFAs of the Italian MPFI-24 confirmed a first-order structure composed of twelve distinct psychological flexibility and inflexibility sub-processes. Additionally, the data supported a second-order model, where global psychological flexibility and inflexibility emerged as two higher-order latent constructs, each encompassing six related first-order sub-processes. Similar results have been reported in other factor analytic validation studies of the MPFI-24 [e.g., Persian (Hekmati et al., 2024); Spanish (Barrado-Moreno et al., 2025)], which similarly tested both models and found the first-order twelve-factor structure to have superior fit. Our results concerning the second-order two-factor structure are also aligned with those of previous studies that only assessed the second-order two-factor structure of the MPFI-24 [original English and French (Grégoire et al., 2020); Chinese (Fang et al., 2024)]. Overall, the factor for the Italian MPFI-24 identified in this study is consistent with the structure established in the original version of the MPFI-24.

The Italian MPFI-24 global psychological flexibility and inflexibility scales demonstrated good internal consistency and their corresponding sub-processes demonstrated reliability coefficients ranging from adequate to excellent (0.71–0.92) and are comparable to those reported for the original English MPFI-24 (0.75–0.92) (Grégoire et al., 2020). Furthermore, the pattern of intercorrelations among the subscales of the Italian MPFI-24 provided evidence for construct validity, demonstrating that while the psychological flexibility and inflexibility global and sub-process scores are correlated in theoretically expected directions, they remain empirically discrete constructs with only moderate overlap. Additionally, measurement invariance analyses confirmed that the Italian MPFI-24 functions consistently across different gender, age, and mental health groups. These psychometric properties align with those of previous MPFI-24 validation studies across cultures (Fang et al., 2024; Hekmati et al., 2024).

## 3. Study 2

Study 2 aimed to reexamine the factorial structure of the Italian MPFI-24 in an independent sample and to evaluate convergent and concurrent validity. Regarding convergent validity, we expected the Italian MPFI-24 global psychological flexibility and sub-processes to be positively and inversely related to other validated measures of psychological flexibility and inflexibility, respectively. We expected the reverse pattern of correlations between the Italian MPFI-24 global psychological inflexibility and sub-processes and other validated measures of psychological flexibility/inflexibility. Further, considering the critical contribution of mindfulness as a component of psychological flexibility, which is proposed to influence all twelve sub-processes in the ACT psychological flexibility framework (Hayes et al., 2012), we also explored correlations between trait mindfulness and MPFI-24 scores to further assess its convergent validity.

Regarding concurrent validity, in line with the ACT psychological flexibility/inflexibility framework, we expected the Italian MPFI-24 global psychological flexibility and sub-process scales to be positively and inversely related to validated measures of psychological distress and well-being, respectively. We expected the reverse pattern of correlations between the Italian MPFI-24 global psychological inflexibility and sub-process scales and validated measures of distress and well-being.

### 3.1. Materials and Methods

#### 3.1.1. Recruitment Procedure and Participants

A total of 503 participants completed an online nationwide survey in Italy. Eligibility criteria included being 18 years or older and fluent in Italian. The survey was developed using Qualtrics platform (Provo, UT, USA) and disseminated via Facebook, WhatsApp, and Instagram. Recruitment was facilitated through advertisements on social networks, combined with a snowball sampling method, from November to December 2023. Response rate could not be determined due to the recruitment methods used. All participation was voluntary, and informed consent was obtained prior to survey completion.

The sample comprised 64.88% females (*n* = 327), with a mean age of 30.94 years (*SD* = 14.07; range 18–86). Most held a high school diploma (*n* = 343; 68.06%), followed by bachelor’s/master’s degrees (*n* = 82; 16.47%) and a middle school diploma (*n* = 51; 10.12%). Regarding employment status, 233 participants (46.32%) were students, 187 (37.18%) were employed, and 83 (16.50%) were unemployed. Almost half of the participants (*n* = 220; 43.74%) were single, while 283 (56.26%) were partnered or married. Regarding socioeconomic status, 30.75% (*n* = 312) reported below-average income, 59.93% (*n* = 302) middle-income, and 9.13% (*n* = 46) above average. Moreover, 11.11% (*n* = 56) of the sample reported that they were currently experiencing psychological or psychiatric challenges, including ongoing therapy and/or medication use.

#### 3.1.2. Measures

Convergent validity of the Italian MPFI-24 was evaluated by examining correlations between the Italian MPFI-24 factors and other established measures of psychological inflexibility/flexibility, as well as a measure of trait mindfulness. The investigation of concurrent validity involved correlations between Italian MPFI-24 scores and those of validated indices of distress and psychological well-being. Measures used to test convergent and concurrent validity are described below.

**Acceptance and Action Questionnaire-II** [AAQ-II; (Bond et al., 2011)]. The AAQ-II assesses psychological inflexibility. Its validated Italian version (Pennato et al., 2013) consists of 10 items rated on a 7-point scale, where 1 indicates “never true” and 7 indicates “always true”. Higher scores indicate greater psychological inflexibility. Observed McDonald’s ω = 0.98.

**Comprehensive Assessment of Acceptance and Commitment Therapy Processes** [CompACT; (Francis et al., 2016)]. Psychological flexibility was assessed by the validated Italian CompACT (Giovannetti et al., 2022), a validated 23-item questionnaire with responses recorded on a 6-point scale, where 1 indicates “strongly disagree” and 6 indicates “strongly agree”. The questionnaire assesses three dimensions: openness to experience, behavioral awareness, and valued action. All items were reverse-coded, meaning higher scores across the total and subscales indicate greater psychological flexibility. The total scale demonstrated a strong internal consistency (observed McDonald’s ω = 0.84), while subscale reliabilities ranged from 0.71 to 0.88.

**Mindful Attention Awareness Scale** [MAAS; (K. W. Brown & Ryan, 2003)]. Trait mindfulness was examined by the validated Italian version of the MAAS (Veneziani & Voci, 2015). This 15-item scale has a 6-point Likert response format where 1 indicates “almost always” and 6 indicates “almost never”. Higher total scores indicate greater mindfulness (observed McDonald’s ω = 0.89).

**Depression, Anxiety, and Stress Scale-21** [DASS-21; (Lovibond & Lovibond, 1995)]. Psychological distress was measured using the validated Italian DASS-21 (Bottesi et al., 2015), which consists of 21 items rated on a 4-point scale, where 0 indicates “did not apply to me at all” and 3 indicates “applied to me very much, or most of the time”. This instrument includes three subdomains: depression, anxiety, and stress. Reliability estimates observed McDonald’s ω varied from 0.87 to 0.91 across the subscales.

**Well-being Numerical Rating Scales** [WB-NRSs; (Bonacchi et al., 2021)]. The WB-NRSs provides a brief multidimensional assessment of well-being. It has five items with each rated on a 10-point scale (Bonacchi et al., 2021). Higher scores indicate increases in well-being. The WB-NRSs evaluates five well-being dimensions: physical, psychological, spiritual, relational, and overall well-being (observed McDonald’s ω = 0.89).

#### 3.1.3. Data Analysis

Most of the analyses were carried out using SPSS version 29. IBM AMOS 26 with the maximum likelihood estimator was employed to reexamine the factor structure of the instrument. We analyzed both the first-order model constituted by the twelve MPFI-24 psychological flexibility and inflexibility sub-processes as first-order factors as well as the second-order model in which psychological flexibility and inflexibility were modeled as two higher-order latent constructs, each defined by their respective six first-order sub-processes. The same fit indices used in Study 1 were employed to evaluate model fit.

The Italian MPFI-24’s convergent validity was examined through Pearson correlations with other measures of psychological flexibility/inflexibility (i.e., AAQ-II and CompACT) as well as a measure of trait mindfulness (i.e., MAAS). To assess concurrent validity, we examined correlations between the Italian MPFI-24 and established measures of psychological distress (i.e., DASS-21) and well-being (i.e., WB-NRS).

### 3.2. Results

#### 3.2.1. Reexamination of the Factorial Structure of the Italian MPFI-24

To reexamine the factor structure of the Italian MPFI-24, we conducted two CFAs (testing the twelve-factor and the two-factor second-order models). The analyses replicated the results of Study 1 with CFAs showing a very good fit (χ^2^ = 395.28, χ^2^/df = 2.04, CFI = 0.97, TLI = 0.95, RMSEA = 0.045 [0.039–0.052], SRMR = 0.043) for the twelve-factor solution, with all loadings significant (*p* < 0.001) and greater than 0.56. Similarly, the two-factor second-order model had a good fit (χ^2^ = 748.77, χ^2^/df = 3.13, CFI = 0.91, TLI = 0.90, RMSEA = 0.065 [0.060–0.070], SRMR = 0.078).

#### 3.2.2. Convergent Validity

As shown in Table 4 and in line with our hypotheses, global psychological flexibility and its six sub-processes were negatively correlated with the AAQ-II, with most coefficients falling in the small to moderate range (*r* = −0.10 to −0.36, *p* < 0.05), thereby supporting convergent validity. Also consistent with expectations, global psychological flexibility and the six psychological flexibility sub-processes exhibited positive correlations with the total score and most subscales of the CompACT (*r* = 0.09 to 0.57, *p* < 0.05). As expected, global psychological inflexibility and five of its sub-processes demonstrated significant positive correlations with the AAQ-II, with moderate to large correlation coefficients (range 0.28–0.55, *p* < 0.01). Nonetheless, the association between experiential avoidance and the AAQ-II was not statistically significant. Consistent with our expectations, global psychological inflexibility and its sub-processes showed significant negative correlations with both the total and subscale scores of the CompACT (range −0.09–−0.67, *p* < 0.05). The only exception was experiential avoidance, which displayed a small significant positive correlation with the valued action subscale of the CompACT (*r* = 0.23, *p* < 0.001).

Finally, statistical analyses confirmed theoretical expectations of relationships between mindfulness and psychological flexibility dimensions. Small to moderate positive correlations (0.14–0.34, *p* < 0.001) were observed between mindfulness and both global psychological flexibility and all its sub-processes. Conversely, comparable negative associations were observed for global psychological inflexibility and five of its sub-processes (range −0.23–−0.30, *p* < 0.001). Experiential avoidance was the only exception, which did not show as significant association with trait mindfulness.

#### 3.2.3. Concurrent Validity

Findings from the correlation analyses assessing concurrent validity are reported in Table 4. As expected, global psychological flexibility and its sub-processes were associated with depression, anxiety, and stress, with small to moderate correlation coefficients (range −0.09–−0.35, *p* < 0.05). However, although acceptance was negatively related to depression, anxiety and stress, only the correlation with depression reached significance. Additionally, global psychological flexibility and all its sub-processes were positively associated with physical, psychological, spiritual, relational, and general well-being, with correlation coefficients ranging from small to moderate in magnitude (range 0.16–0.39, *p* < 0.001).

In contrast, and consistent with expectations, global psychological inflexibility and its sub-processes showed positive associations with depression, anxiety, and stress, with correlation coefficients ranging from small to large (0.12–0.46, *p* < 0.001). The sole exception was experiential avoidance, which showed no significant association with depression and anxiety. Additionally, global psychological inflexibility and five of its sub-processes were negatively associated with all well-being dimensions, also evidencing correlation coefficients in the small to moderate range (−0.10–−0.38, *p* < 0.001). The only exception was experiential avoidance, which displayed small positive correlations with physical, psychological, spiritual, and relational well-being (range −0.11–−0.13, *p* < 0.001), and a non-significant correlation with general well-being.

### 3.3. Discussion

Factor analyses of the Italian MPFI-24 in an independent sample replicated the factor structure obtained in Study 1. Specifically, results demonstrated a very good fit for the first-order model, which included the twelve psychological flexibility and inflexibility sub-processes. Additionally, the second-order model, where global psychological flexibility and inflexibility served as two second-order latent variables with their corresponding six sub-processes as first-order latent variables, also showed a good fit.

The Italian MPFI-24 demonstrated strong convergent validity. The instrument displayed associations with other scales of psychological flexibility/inflexibility and with trait mindfulness in directions that were consistent with the ACT psychological flexibility framework. Concurrent validity was also supported, as psychological flexibility and inflexibility global and subscale scores showed associations with indicators of distress and well-being that were consistent with theoretical predictions. However, one notable exception was experiential avoidance which evidenced unexpected correlations in both convergent and concurrent validity analyses. The convergent validity analysis revealed that experiential avoidance was not significantly linked to the AAQ-II and trait mindfulness and exhibited a small positive correlation with the valued action subscale of the CompACT. Regarding concurrent validity, it was only positively related to stress (but not to depression and anxiety), and displayed negative associations with all well-being dimensions. Nevertheless, similar unexpected results regarding experiential avoidance have been observed in the Italian validation study of the parent MPFI (Landi et al., 2021b), and validation studies of the MPFI-24 (Hekmati et al., 2024; Sundström et al., 2023; Tabrizi et al., 2023).

## 4. General Discussion

The present research comprised two studies designed to validate the Italian MPFI-24 by assessing its factor structure, reliability, validity, and measurement invariance using two general population samples. Factor analyses of the Italian MPFI-24 in both studies provided robust support for its first-order twelve-factor structure and acceptable support for the two-factor hierarchical model. These findings align with previous factor analytic studies of the MPFI-24, which likewise identified that both a two-factor second-order model (Fang et al., 2024; Grégoire et al., 2020) and a twelve-factor first-order model (Barrado-Moreno et al., 2025; Hekmati et al., 2024) as the best fitting solution. Notably, in research evaluating both models, each model was reported to provide good or adequate fit (Barrado-Moreno et al., 2025; Hekmati et al., 2024).

These findings affirm that the factor structure of the Italian MPFI-24 is consistent with the ACT psychological flexibility framework. While the twelve sub-processes are interconnected, each one is distinctive and as such, they may not simply exist on a continuum but interact in more complex ways that influence therapeutic outcomes or research findings [e.g., (Pakenham et al., 2023; Rogge & Lin, 2024; Stabbe et al., 2019)]. Overall, the Italian MPFI-24 serves as a multidimensional measure consisting of twelve distinct scales organized into two higher-order dimensions, which offers a robust measurement instrument for assessing individual variations in psychological flexibility/inflexibility (Rogge & Lin, 2024; Tabrizi et al., 2023).

Results of Study 1 showed that the Italian MPFI-24 global psychological flexibility and inflexibility scales and their respective subscales displayed good internal consistency, evidenced by reliability coefficients ranging from 0.71 to 0.92. These reliability coefficients are similar to those reported for the English MPFI-24 (0.75–0.92) (Grégoire et al., 2020). Furthermore, the intercorrelations among the Italian MPFI-24 subscales showed moderate but not complete overlap, evidencing their interconnectedness and distinctiveness.

Study 1 results also supported the measurement invariance of the Italian MPFI-24 across gender, age, and mental health status, suggesting consistent performance of the instrument across these dimensions. These findings align with those reported in previous validation studies of the MPFI-24 (Fang et al., 2024; Hekmati et al., 2024) as well as validation studies of the parent MPFI (Landi et al., 2021b; Lin et al., 2020; Tabrizi et al., 2023). This evidence indicates that the Italian MPFI-24 allows for reliable comparisons of latent means among groups differentiated by gender, age, and mental health status within the Italian context.

In terms of additional psychometric evidence from Study 2, the Italian MPFI-24 demonstrated convergent validity through small to large correlations between global psychological flexibility and inflexibility (as well as most of their sub-processes) and established measures of flexibility/inflexibility (i.e., AAQ-II and CompACT), as well as trait mindfulness (i.e., MAAS), which were aligned with theoretical expectations. These findings are consistent with those form validation studies of the parent MPFI (Rolffs et al., 2018) and the MPFI-24 (Barrado-Moreno et al., 2025; Fang et al., 2024; Grégoire et al., 2020; Hekmati et al., 2024). Additionally, concurrent validity was evidenced by significant theoretically consistent relationships between the global psychological flexibility and inflexibility scales and most of their sub-processes with measures of distress and well-being. These results align with those from studies of the parent MPFI (Landi et al., 2021b; Rolffs et al., 2018) and the MPFI-24 (Fang et al., 2024; Hekmati et al., 2024).

Interestingly, experiential avoidance evidenced unexpected associations with some MPFI-24 subscales and other measures in validity analyses. Contrary to the psychological flexibility framework, experiential avoidance showed no statistically significant associations with four psychological inflexibility sub-processes and exhibited small but significant positive correlations with three psychological flexibility sub-processes. In terms of convergent validity, contrary to expectations, experiential avoidance did not show significant correlations with the AAQ-II and trait mindfulness but exhibited a small positive correlation with the CompACT valued action subscale, which assesses a facet of psychological flexibility. Regarding concurrent validity, unexpectedly experiential avoidance did not show significant negative correlations with depression or anxiety, although it was negatively correlated with stress. Also unexpectedly, it manifested significant but weak positive correlations with some well-being dimensions (i.e., physical, psychological, spiritual, and relational).

These unexpected experiential avoidance findings are consistent with those from previous validation studies of the MPFI-24. Specifically, similar to our findings, the Spanish MPFI-24 validation study reported that experiential avoidance was only significantly related to some psychological inflexibility sub-processes, and was positively associated with one psychological flexibility sub-process (Barrado-Moreno et al., 2025). Furthermore, as for convergent validity, experiential avoidance was not significantly associated with other measures of psychological flexibility/inflexibility in either the Spanish or French MPFI-24 validation studies (Barrado-Moreno et al., 2025; Grégoire et al., 2020). Notably, the Spanish MPFI-24 study found an unexpected positive association between experiential avoidance and the AAQ-II (Barrado-Moreno et al., 2025). Finally, our concurrent validity findings regarding experiential avoidance align with those studies that investigated the French and Spanish MPFI-24, which found that experiential avoidance was unrelated to measures of distress and well-being (Barrado-Moreno et al., 2025; Grégoire et al., 2020). Several explanations have been proposed in the literature for the abovementioned pattern of associations between experiential avoidance and other MPFI assessed psychological flexibility/inflexibility sub-processes and standardized psychosocial outcome measures that are inconsistent with the ACT psychological flexibility framework. First, the wording of some MPFI experiential avoidance items, originally adapted from mindfulness-based scales [e.g., Philadelphia Mindfulness Scale; (Cardaciotto et al., 2008)], might not be sufficiently specific for capturing experiential avoidance as intended (Grégoire et al., 2020; Tabrizi et al., 2023). Second, translation challenges across languages (e.g., Italian, French, Spanish, Swedish) could obscure accurate interpretation of experiential avoidance items (Barrado-Moreno et al., 2025; Grégoire et al., 2020; Tabrizi et al., 2023). Third, experiential avoidance is likely to entail context-sensitive adaptiveness, especially during acute crises or major societal disruptions such as the COVID-19 pandemic. In these contexts, temporary experiential avoidance can provide immediate emotional relief and short-term coping benefits (Landi et al., 2021b; Pakenham et al., 2020, 2023). Indeed, person-centered analyses have identified profiles wherein high experiential avoidance co-occurs with high psychological flexibility, highlighting nuanced response patterns overlooked in other variable-centered studies (Pakenham et al., 2023; Stabbe et al., 2019). Finally, variability in how experiential avoidance relates to other theoretically relevant variables may reflect measurement limitations. For instance, the AAQ-II—commonly used to assess experiential avoidance—has been shown to overlap with psychological distress (Doorley et al., 2020; Landi et al., 2021a).

Beyond these findings, the present results also highlight the practical value of the MPFI-24 as it retains assessment of the 12 ACT-consistent sub-processes while making multidimensional assessment more feasible in settings where repeated or broader assessment batteries are required, thereby addressing an important gap left by shorter measures that do not simultaneously capture psychological flexibility, psychological inflexibility, and their component processes. This is especially relevant in research designs involving repeated measurement and within-person processes, including ecological momentary assessment applications (Fraser et al., 2026; Krafft et al., 2025), as well as in clinical contexts where clinicians may need a brief but theoretically informative instrument for profiling psychological flexibility/inflexibility processes and monitoring change over time (Krafft et al., 2025; Pakenham et al., 2023).

This study has several limitations. Participants were recruited through convenience sampling, and both studies were characterized by an overrepresentation of female respondents, while Study 1 also included a high proportion of university-educated participants. These sample characteristics may restrict the generalizability of the findings. At the same time, the replication of the factor structure across Study 1 and Study 2, despite differences in educational composition, and the support for measurement invariance across gender in Study 1 suggest that the internal structure of the Italian MPFI-24 was stable across these sample characteristics. Specifically, the gender invariance results indicate that the instrument functioned similarly for men and women, supporting the reliable comparison of MPFI-24 latent means across gender groups in the Italian population. Furthermore, all data were collected via online self-report measures, increasing the potential for common method variance. Additionally, the cross-sectional design precluded evaluation of the test–retest reliability of the Italian MPFI-24 and its sensitivity to changes across time. Future studies should utilize longitudinal designs and recruit samples that better reflect the broader population. A further limitation relates to the estimation method. Although the 6-point MPFI-24 items were treated as approximately continuous and analyzed using maximum likelihood estimation, they represent ordered response categories. Future studies should therefore examine whether the present findings replicate when estimators designed for ordinal data, such as weighted least squares mean and variance adjusted, are used instead (Rhemtulla et al., 2012; Robitzsch, 2020). Additionally, although our studies included subgroups of participants who reported current mental health difficulties and/or receiving ongoing psychological or pharmacological treatment, future research should test the psychometric proprieties of the Italian MPFI-24 in populations with independently diagnosed mental disorders. Such studies would identify which psychological flexibility/inflexibility sub-processes are most closely associated with therapeutic outcomes in specific diagnostic groups and, more broadly, in relation to health and well-being.

## 5. Conclusions

Overall, the present findings support the reliability and validity of the Italian MPFI-24 for assessing global psychological flexibility and inflexibility and their corresponding sub-processes within the Italian context. Given its brevity, the MPFI-24 is especially suited to research and clinical contexts where assessment time is limited, offering substantial efficiency advantages compared to the original, longer parent MPFI. In research endeavors, the MPFI-24 could reduce participant burden, enhance retention rates, and facilitate large-scale projects, including daily diary studies or ecological momentary assessments. In clinical applications, its concise format is particularly suited to repeated, routine screenings for efficiently tracking of client progress without excessive demands on clients or clinicians.

## Figures and Tables

**Table 1:** Fit indices for the factorial structure of the Italian MPFI-24 (N = 1693).

Model	χ^2^	χ^2^/*df*	CFI	TLI	RMSEA [90% CI]	SRMR
One-factor model	12,971.031 ***	51.472	0.496	0.448	0.173 [0.170–0.175]	0.122
Two-factor model	10,140.187 ***	40.399	0.608	0.569	0.153 [0.150–0.155]	0.100
**Twelve-factor model**	565.714 ***	**2.916**	**0.985**	**0.979**	**0.034 [0.030–0.037]**	**0.059**
One-factor second-order model	2634.415 ***	10.977	0.905	0.891	0.077 [0.074–0.079]	0.071
Two-factor second-order model	1701.942 ***	7.121	0.942	0.933	0.060 [0.057–0.063]	0.063

Note. CFI = Comparative Fit Index; TLI = Tucker–Lewis Index; RMSEA = Root Mean Square Error of Approximation; SRMR = Standardized Root Mean Square Residuals. CI = confidence interval. *** *p* < 0.001. Bold indicates the best-fitting model.

**Table 2:** Factor loadings and reliability indices of the Italian MPFI-24 (N = 1693).

Factor	β	ω (95% CI)	Factor	β	ω (95% CI)
**Psychological flexibility**			**Psychological inflexibility**		
*Acceptance*			*Experiential avoidance*		
MPFI 1	0.690 ***	0.707 (0.668–0.741)	MPFI 13	0.935 ***	0.863 (0.846–0.879)
MPFI 2	0.791 ***		MPFI 14	0.822 ***	
*Contact with present moment*			*Lack of contact with present moment*		
MPFI 3	0.852 ***	0.837 (0.814–0.858)	MPFI 15	0.903 ***	0.869 (0.852–0.885)
MPFI 4	0.851 ***		MPFI 16	0.861 ***	
*Self-as-context*			*Self-as-content*		
MPFI 5	0.782 ***	0.797 (0.771–0.821)	MPFI 17	0.886 ***	0.874 (0.858–0.889)
MPFI 6	0.851 ***		MPFI 18	0.892 ***	
*Defusion*			*Fusion*		
MPFI 7	0.915 ***	0.905 (0.891–0.918)	MPFI 19	0.919 ***	0.897 (0.884–0.909)
MPFI 8	0.905 ***		MPFI 20	0.889 ***	
*Contact with values*			*Lack of contact with values*		
MPFI 9	0.740 ***	0.740 (0.707–0.770)	MPFI 21	0.824 ***	0.820 (0.798–0.841)
MPFI 10	0.777 ***		MPFI 22	0.836 ***	
*Committed action*			*Inaction*		
MPFI 11	0.806 ***	0.844 (0.823–0.864)	MPFI 23	0.913 ***	0.914 (0.902–0.925)
MPFI 12	0.903 ***		MPFI 24	0.935 ***	

Note. β = standardized factor loading; SE = standard error; *** *p* < 0.001; ω = McDonald omega; CI = confidence interval.

**Table 3:** Intercorrelations among the Italian MPFI-24 global psychological flexibility and inflexibility scales and sub-processes (N = 1693).

	*M* (*SD*)	1	1a	1b	1c	1d	1e	1f	2	2a	2b	2c	2d	2e
1. Global Psychological Flexibility	3.76 (0.82)	-												
1a. Acceptance	3.42 (1.03)	0.682 ***	-											
1b. Present Moment Awareness	3.97 (1.13)	0.674 ***	0.414 ***	-										
1c. Self-as-context	3.75 (1.13)	0.808 ***	0.503 ***	0.463 ***	-									
1d. Defusion	3.30 (1.17)	0.759 ***	0.473 ***	0.317 ***	0.605 ***	-								
1e. Values	4.09 (1.09)	0.743 ***	0.322 ***	0.416 ***	0.486 ***	0.454 ***	-							
1f. Committed Action	4.04 (1.14)	0.757 ***	0.346 ***	0.376 ***	0.510 ***	0.487 ***	0.620 ***	-						
2. Global Psychological Inflexibility	2.98 (0.85)	−0.522 ***	−0.327 ***	−0.279 ***	−0.387 ***	−0.538 ***	−0.400 ***	−0.372 ***	-					
2a. Experiential avoidance	3.58 (1.16)	0.020	−0.151 ***	−0.054 *	0.052 *	0.033	0.112 ***	0.085 ***	0.318 ***	-				
2b. Lack of contact with present moment	2.85 (1.19)	−0.357 ***	−0.179 ***	−0.307 ***	−0.256 ***	−0.261 ***	−0.310 ***	−0.265 ***	0.665 ***	0.122 ***	-			
2c. Self-as-content	2.93 (1.14)	−0.354 ***	−0.244 ***	−0.186 ***	−0.267 ***	−0.416 ***	−0.221 ***	−0.225 ***	0.757 ***	0.188 ***	0.384 ***	-		
2d. Fusion	3.15 (1.37)	−0.433 ***	−0.247 ***	−0.132 ***	−0.355 ***	−0.572 ***	−0.229 ***	−0.296 ***	0.784 ***	0.29	0.370 ***	0.536 ***	-	
2e. Lack of contact with values	2.73 (1.14)	−0.430 ***	−0.198 ***	−0.239 ***	−0.291 ***	−0.329 ***	−0.468 ***	−0.377 ***	0.682 ***	−0.010	0.450 ***	0.370 ***	0.439 ***	-
2f. Inaction	2.63 (1.33)	−0.526 ***	−0.289 ***	−0.218 *	−0.415 ***	−0.562 ***	−0.424 ***	−0.408 ***	0.797 ***	0.026	0.391 ***	0.494 ***	0.699 ***	0.539 ***

Note. * *p* < 0.05, *** *p* < 0.001.

**Table 4:** Correlations among the Italian MPFI-24 global psychological flexibility and inflexibility scales and sub-processes, other measures of psychological flexibility and inflexibility, mindfulness, psychological distress, and well-being (N = 503).

	AAQ-II	CopACT Openness to Experience	CopACT Behavioral Awareness	CopACT Valued Action	CompACT Total	MASS Mindfulness	DASS-21 Depression	DASS-21 Anxiety	DASS-21 Stress	WB-NRSs Physical	WB-NRSs Psychological	WB-NRSs Spiritual	WB-NRSs Relational	WB-NRSs General
1. Global Psychological Flexibility	−0.340 ***	0.221 ***	0.251 ***	0.571 ***	0.476 ***	0.347 ***	−0.300 ***	−0.236 ***	−0.192 ***	0.256 ***	0.405 ***	0.390 ***	0.355 ***	0.354 ***
1a. Acceptance	−0.104 *	0.142 **	−0.026	0.283 ***	0.187 ***	0.148 ***	−0.095 *	−0.074	−0.048	0.159 ***	0.229 ***	0.183 ***	0.168 ***	0.125 **
1b. Present Moment Awareness	−0.209 ***	0.172 ***	0.217 ***	0.384 ***	0.354 ***	0.246 ***	−0.167 ***	−0.152 ***	−0.092 *	0.160 ***	0.257 ***	0.254 ***	0.229 ***	0.231 ***
1c. Self-as-context	−0.259 ***	0.164 ***	0.192 ***	0.445 ***	0.365 ***	0.260 ***	−0.256 ***	−0.209 ***	−0.157 ***	0.194 ***	0.304 ***	0.300 ***	0.243 ***	0.298 ***
1d. Defusion	−0.361 ***	0.230 ***	0.180 ***	0.368 ***	0.360 ***	0.296 ***	−0.348 ***	−0.232 ***	−0.339 ***	0.189 ***	0.381 ***	0.342 ***	0.351 ***	0.335 ***
1e. Values	−0.271 ***	0.131 **	0.257 ***	0.517 ***	0.408 ***	0.296 ***	−0.238 ***	−0.173 ***	−0.089 *	0.239 ***	0.318 ***	0.324 ***	0.290 ***	0.293 ***
1f. Committed Action	−0.318 ***	0.155 ***	0.303 ***	0.580 ***	0.467 ***	0.317 ***	−0.240 ***	−0.219 ***	−0.125 **	0.256 ***	0.337 ***	0.352 ***	0.312 ***	0.308 ***
2. Global Psychological Inflexibility	0.539 ***	−0.549 ***	−0.620 ***	−0.247 ***	−0.669 ***	−0.306 ***	0.457 ***	0.376 ***	0.378 ***	−0.170 ***	−0.321 ***	−0.316 ***	−0.270 ***	−0.281 ***
2a. Experiential avoidance	0.027	−0.288 ***	−0.095 *	0.233 ***	−0.091 *	0.013	0.080	0.034	0.124 **	0.107 *	0.133 **	0.114 *	0.130 **	0.084
2b. Lack of contact with present moment	0.282 ***	−0.399 ***	−0.571 ***	−0.165 ***	−0.533 ***	−0.256 ***	0.277 ***	0.277 ***	0.229 ***	−0.100 *	−0.157 ***	−0.165 ***	−0.183 ***	−0.189 ***
2c. Self-as-content	0.441 ***	−0.484 ***	−0.442 ***	−0.188 ***	−0.511 ***	−0.307 ***	0.385 ***	0.294 ***	0.338 ***	−0.146 **	−0.265 ***	−0.271 ***	−0.225 ***	−0.208 ***
2d. Fusion	0.559 ***	−0.484 ***	−0.456 ***	−0.203 ***	−0.544 ***	−0.253 ***	0.451 ***	0.379 ***	0.406 ***	−0.204 ***	−0.364 ***	−0.319 ***	−0.311 ***	−0.327 ***
2e. Lack of contact with values	0.426 ***	−0.333 ***	−0.556 ***	−0.326 ***	−0.562 ***	−0.256 ***	0.328 ***	0.288 ***	0.203 ***	−0.152 ***	−0.275 ***	−0.276 ***	−0.193 ***	−0.226 ***
2f. Inaction	0.523 ***	−0.401 ***	−0.512 ***	−0.352 ***	−0.590 ***	−0.232 ***	0.406 ***	0.313 ***	0.301 ***	−0.200 ***	−0.391 ***	−0.383 ***	−0.327 ***	−0.299 ***

Note. * *p* < 0.05, ** *p* < 0.01, *** *p* < 0.001.

## Data Availability

The dataset analyzed during the current study is available from the corresponding authors upon reasonable request.

## Supplementary Materials

The following supporting information can be downloaded at: https://www.mdpi.com/article/10.3390/bs16040510/s1.
