# Supplementary material for: Validation of the Italian Multidimensional Psychological Flexibility Inventory Short Form (MPFI-24)
Source: Behav Sci (Basel). 2026 Mar 29;16(4):510. doi: 10.3390/bs16040510 (PMC13112982; doi:10.3390/bs16040510)
Supplement: Supplementary file 1 [file behavsci-16-00510-s001.zip › behavsci-4177655-supplementary.pdf]

Supplementary Materials

Table S1

Descriptive Data for the Italian MPFI-24 (N=1,693).

Item	Mean (*SD*)	Skewness	Kurtosis
MPFI 1	3.29 (1.13)	0.354	-0.120
MPFI 2	3.56 (1.22)	0.056	-0.460
MPFI 3	4.11 (1.19)	-0.206	-0.412
MPFI 4	3.84 (1.24)	-0.043	-0.514
MPFI 5	3.77 (1.20)	-0.058	-0.434
MPFI 6	3.73 (1.27)	-0.066	-0.505
MPFI 7	3.36 (1.23)	0.211	-0.533
MPFI 8	3.25 (1.21)	0.279	-0.426
MPFI 9	4.11 (1.19)	-0.231	-0.471
MPFI 10	4.06 (1.22)	-0.184	-0.475
MPFI 11	4.02 (1.24)	-0.190	-0.509
MPFI 12	4.07 (1.20)	-0.214	-0.392
MPFI 13	3.59 (1.27)	0.053	-0.610
MPFI 14	3.57 (1.19)	0.155	-0.443
MPFI 15	2.98 (1.31)	0.363	-0.563
MPFI 16	2.72 (1.22)	0.575	-0.287
MPFI 17	2.94 (1.46)	0.346	-0.829
MPFI 18	2.92 (1.49)	0.390	-0.822
MPFI 19	3.22 (1.39)	0.326	-0.665
MPFI 20	3.07 (1.48)	0.361	-0.852
MPFI 21	2.70 (1.20)	0.636	0.101
MPFI 22	2.75 (1.27)	0.577	-0.239
MPFI 23	2.65 (1.38)	0.645	-0.374
MPFI 24	2.61 (1.38)	0.706	-0.315

Table S2

Italian MPFI-24 Measurement Invariance Across Gender, Age, and Mental Health Status (N = 1,693).

	Model	χ^2	Δdf	CFI	ΔCFI	TLI	ΔTLI	RMSEA	ΔRMSEA
Gender	Configural	835.89	-	.982	-	.975	-	.026	-
	Metric	865.03	12	.981	.001	.974	.001	.026	.000
	Scalar	945.00	24	.979	.002	.973	.001	.027	.001
	Residual	1114.85	94	.976	.003	.975	.002	.026	-.001
Age	Configural	863.35	-	.981	-	.973	-	.027	-
	Metric	874.83	12	.981	.000	.974	.001	.027	.000
	Scalar	1113.93	24	.972	.009	.964	-.01	.031	.004
	Residual	1338.32	94	.967	.005	.965	.001	.031	.000
Mental Health Status	Configural	828.79	-	.982	-	.975	-	.026	-
	Metric	842.82	12	.982	.000	.975	.000	.026	.000
	Scalar	1009.25	24	.976	.006	.969	.006	.029	.003
	Residual	1263.27	94	.970	.006	.968	.001	.029	.000

Note. CFI = Comparative Fit Index; TLI = Tucker-Lewis Index; RMSEA = Root Mean Square Error of Approximation; The deltas (Δ) represent the change between the levels of measurement invariance.
